# Mitochondrial calcium overload contributes to cannabinoid‐induced paraptosis in hormone‐responsive breast cancer cells

**DOI:** 10.1111/cpr.13650

**Published:** 2024-05-09

**Authors:** A. de la Harpe, N. Beukes, C. Frost

**Affiliations:** ^1^ Department of Biochemistry and Microbiology Nelson Mandela University Port Elizabeth South Africa

## Abstract

Studies have shown that natural products can induce paraptosis in tumour cell lines. Paraptosis is characterized by cytoplasmic vacuolation arising from the endoplasmic reticulum (ER) and mitochondria. The mechanism of paraptosis is unclear; however, dysregulation of Ca^2+^ homeostasis is believed to affect paraptosis induction. This study investigated the mechanism of cell death induced by a phytocannabinoid ratio in the MCF7 breast cancer cell line. The crystal violet assay was used to detect changes in viability and morphology changes were investigated using light and transmission electron microscopy. Various inhibitors, fluorescent staining with high‐content screening, and Western blot analysis were used to investigate different cell death mechanisms. The phytocannabinoid ratio induced significant cell death and cytoplasmic vacuolation in MCF7 cells; however, no apoptosis, necrosis, autophagy, or ferroptosis was detected. Vacuolation induced by phytocannabinoid treatment was inhibited by cycloheximide, suggesting paraptosis induction. The mechanism of paraptosis induction was investigated, and it was found that treatment (1) induced ER dilation and mitochondrial swelling, (2) induced significant ER stress and mitochondrial Ca^2+^ overload and dysfunction, which appeared to be mediated by the voltage‐dependent anion channel, and (3) significantly impaired all mitochondrial metabolic pathways. The data demonstrated that paraptosis induced by the cannabinoid ratio was mediated by Ca^2+^ flux from the ER to the mitochondria. These findings highlight a novel mechanism of cannabinoid‐induced cell death and emphasize the anti‐cancer potential of cannabinoid ratios, which exhibited enhanced effects compared to individual cannabinoids.

## Graphical abstract

Treatment with phytocannabinoids in breast cancer cells induces significant calcium flux from the endoplasmic reticulum (ER) to the mitochondria via voltage‐dependent anion channel, causing significant ER stress and mitochondrial calcium overload, ER and mitochondrial swelling, disruption to mitochondrial structure and function, and ultimately the induction of paraptosis in MCF7 cells.

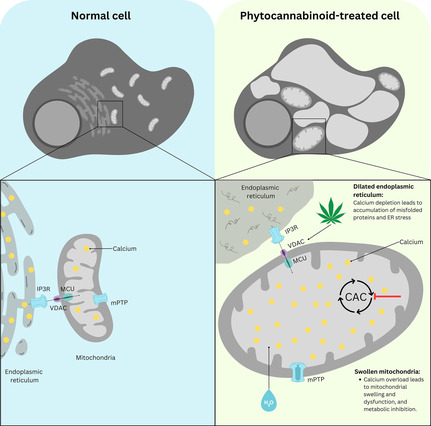

## 1 INTRODUCTION

With an estimated 2.3 million cases in 2020, breast cancer accounted for approximately 1 in 4 of all cancer cases in women.^1^ More than 70% of invasive breast cancers are hormone receptor‐positive.^2^ Endocrine therapy is the standard of treatment for these types of cancers, with chemotherapy reserved for more advanced or severe cases.^3^ However, both hormone therapy and chemotherapy have long‐lasting toxic side effects that significantly decrease the patient's quality of life.^4^

In recent years, medicinal plants have become increasingly popular due to their favourable safety profiles, efficacy and lack of significant adverse effects.^5^ One such plant is *Cannabis*, a well‐known member of the *Cannabaceae* family.^6^ Phytocannabinoids account for more than 100 of the 400 known chemical compounds in *Cannabis* plants and mimic the effects of endogenous cannabinoids by activating receptors in the endocannabinoid system.^7^ The most well‐known cannabinoid receptors are the CB1 and CB2 receptors, discovered in 1988 and 1993, respectively.^8^, ^9^ In addition to these receptors, cannabinoids have been reported to interact with and modulate the activity of numerous other receptors, including members of the transient receptor potential (TRP) cation channel family, peroxisome proliferator‐activated receptors (PPAR) α and γ, and various orphan G‐protein coupled receptors.^10^, ^11^

In the context of cancer, cannabinoids have primarily been used for their palliative effects to treat the side effects of chemotherapy; however, their tumour‐suppressive properties have been known since the first observation of their antitumor effects in 1975.^12^ The major cannabinoid‐induced antitumor effects  observed are the induction of cell cycle arrest, endoplasmic reticulum (ER) stress, autophagy, and apoptosis, as well as the inhibition of angiogenesis, invasion, metastasis, and growth factor expression.^13^, ^14^, ^15^, ^16^ However, variability in the antitumor effects of *Cannabis* plant extracts is often observed, primarily due to differences in the phytochemical composition.^17^ Therefore, standardization of cannabis use as an anticancer agent may be limited to purified or chemically synthesized cannabinoids, as opposed to *Cannabis* plant extracts.

The toxicity of currently used therapies and the well‐known ability of cancer cells to evade apoptosis and acquire resistance to treatment have highlighted the need for less toxic cancer therapies that induce alternative mechanisms of cell death. Paraptosis is one of these potential mechanisms. Paraptosis is biochemically and morphologically distinct from apoptosis and is characterized by cytoplasmic vacuolation arising from the ER and mitochondria as well as a dependence on protein translation.^18^ Various mechanisms of paraptosis induction have been described, including (1) ER stress, (2) calcium (Ca^2+^) overload in the mitochondria, (3) proteasomal inhibition, (4) opening of ion channels, (5) generation of reactive oxygen species (ROS) and (6) insulin growth factor 1 receptor expression.^19^ Many natural products have been shown to induce paraptosis by various mechanisms^20^, ^21^ and a synthetic cannabinoid (WIN55 212‐2) was shown to induce paraptosis independent of cannabinoid receptor signalling.^22^

A phytocannabinoid combination (C6 ratio) optimized by Schoeman et al.^23^ resulted in significant cell death and extensive cytoplasmic vacuolation in MCF7 cells, suggesting paraptosis induction. This study aimed to identify and characterize the mechanism of cell death induced by the phytocannabinoid ratio in the hormone‐responsive MCF7 breast cancer cell line.

## 2 MATERIALS AND METHODS

### 2.1 Cell culture conditions

The MCF7 (ECACC no. 86012803) and MCF10A (kindly donated by Prof. A. Edkins) cell lines were used in this study. MCF7 cells were maintained in high‐glucose DMEM (Biowest) supplemented with 10% fetal bovine serum (FBS, Capricorn Scientific). MCF10A cells were maintained in high‐glucose DMEM:Ham F12 (Biowest) supplemented with 5% v/v donor horse serum (Biowest), 10 μg/mL insulin (Sigma‐Aldrich), 0.5 μg/mL hydrocortisone (Sigma‐Aldrich), and 20 ng/mL human epidermal growth factor (Peprotech). Cells were maintained in a humidified incubator at 37°C and 5% CO_2_.

### 2.2 Treatment and controls

MCF7 and MCF10A cells were seeded in 96 well plates at densities of 6000 cells/well and 15,000 cells/well, respectively. Cells were treated with various concentrations of the C6 ratio which contained Δ^9^‐tetrahydrocannabinol (THC), cannabigerol (CBG), cannabinol (CBN) (RESTEK), and cannabidiol (CBD, LGC) in dimethyl sulfoxide (DMSO). As per patent number EP4203925A1,^24^ the constant ratio of cannabinoids in the C6 ratio is 3.7:1:1.9:4.8 for THC:CBG:CBN:CBD. The total concentrations of the C6 ratio used in this study were 27, 33, and 39 μM and were based on the IC_50_, IC_75,_ and IC_90_ values of the C6 ratio, respectively, as determined by Schoeman et al.^23^ after 48 h of treatment. These concentrations refer to the combined molar concentration of [THC] (μM) + [CBG] (μM) + [CBN] (μM) + [CBD] (μM). A DMSO vehicle control was included and corresponded to the DMSO concentration in the highest concentration of the ratio (39 μM = 0.23% DMSO).

### 2.3 Cell viability

Changes in viability were measured using crystal violet staining as described by Feoktistova et al.^25^ Spent medium was removed and cells were washed using distilled water. Cells were stained with the crystal violet staining solution (0.5% w/v crystal violet [Sigma‐Aldrich] in 20% v/v methanol) for 20 min with gentle agitation and washed under a stream of water until the excess stain was removed. Once dry, methanol was added to solubilize the bound crystal violet for 20 min with gentle agitation. The absorbance (570 nm) was measured using a BioTek Epoch2 microplate reader.

### 2.4 Fluorescent staining, imaging, and analysis

#### 2.4.1 Image acquisition and analysis

Nine images per well were acquired using an ImageXpress Micro XLS Widefield High‐Content Analysis System (Molecular Devices) with a 10× objective. The images were analysed using the MetaXpress® High‐Content Image Acquisition & Analysis Software.

#### 2.4.2 FITC Annexin V/Dead Cell Apoptosis Kit

For cell cycle analysis, cells were stained with FITC Annexin V (Invitrogen™ by Thermo Fisher Scientific) (1:20 dilution) and 2 μg/mL bisbenzimide H 33342 trihydrochloride (Hoechst, Sigma‐Aldrich) in 1× binding buffer for 30 min at 37°C. Images were acquired using FITC (excitation/emission 498/517 nm) and DAPI (excitation/emission 359/461 nm) filters. Images were analyed using the cell cycle application module which generates various parameters including the count and percentage of cells in the G0/G1, S, G2, early M, and late M phases.

For apoptosis/necrosis detection, cells stained with FITC Annexin V and Hoechst were stained with propidium iodide (PI) at a final concentration of 1 μg/mL. Images were acquired using FITC, DAPI, and Texas Red (excitation/emission 596/615 nm) filters.

#### 2.4.3 C11‐BODIPY™ 581/591 nm

Cells were stained with 10 μM C11‐BODIPY™ 581/591 (Invitrogen™ by Thermo Fisher Scientific) and 2 μg/mL Hoechst in Dulbecco's phosphate‐buffered saline with Ca^2+^ and Mg^2+^ (DPBS) for 30 min at 37°C. Images were acquired using FITC, DAPI, and Texas Red filters.

#### 2.4.4 LysoTracker™ green

Cells were stained with 50 nM LysoTracker™ (Invitrogen™ by Thermo Fisher Scientific) and 2 μg/mL Hoechst in DPBS for 30 min at 37°C. Images were acquired using FITC and DAPI filters.

#### 2.4.5 CytoPainter Orange mitochondrial stain

Cells were stained with 1× CytoPainter (Abcam) and 2 μg/mL Hoechst in live cell staining buffer for 30 min at 37°C. Images were acquired using TRITC (excitation/emission, 557/576 nm) and DAPI filters.

#### 2.4.6 Nonyl acridine orange

Cells were stained with 1 μM nonyl acridine orange (NAO, Invitrogen™ by Thermo Fisher Scientific) and 2 μg/mL Hoechst in DPBS for 30 min at 37°C. Images were acquired using FITC and DAPI filters.

#### 2.4.7 CellROX™ orange

The cells were stained with 2.5 μM CellROX™ Orange (Invitrogen™ by Thermo Fisher Scientific) and 2 μg/mL Hoechst in DPBS for 30 min at 37°C. Images were acquired using TRITC and DAPI filters.

#### 2.4.8 Rhod‐2 AM

Cells were stained with 5 μM Rhod‐2 AM^26^ (Abcam) and 2 μg/mL Hoechst in DPBS for 30 min at 37°C. Images were acquired using TRITC and DAPI filters.

### 2.5 Transmission electron microscopy

Cells were fixed in 2.5% glutaraldehyde and post‐fixed in 1% w/v osmium tetroxide in 0.1 M phosphate buffer. They were then washed and dehydrated before being embedded in resin. The samples were polymerized at 60°C for 36 h before an ultramicrotome was used to cut ultrathin sections stained with uranyl acetate and counter‐stained with lead citrate.

### 2.6 SDS‐PAGE and western blot analysis

Cell lysates were prepared using lysis buffer (50 mM TRIS, 2 mM EDTA, 0.1% Triton X‐100) and sonication for 30 s. Protein was quantified using the Bradford protein assay^27^ and equal protein concentrations were precipitated by adding acetone (1:2 lysate: acetone) at −20°C for 90 min. The solutions were centrifuged (12,045 × *g*) for 10 min, the supernatant was discarded, and the pellet dried. Sample buffer was added and samples were incubated overnight at 4°C.

Proteins were resolved on SDS‐PAGE on duplicate gels. One gel was stained with Coomassie blue and proteins from the duplicate gel were transferred to a PVDF membrane using the Bio‐Rad Semi‐dry Trans‐Blot Turbo transfer system (20 V for 20 min). After blocking overnight with 5% w/v skimmed milk powder, the membrane was washed 3× and probed with the primary GRP78 (Santa Cruz Biotechnology, sc‐13968) for 3 h or CHOP (Cell Signaling Technology, D46F1) overnight. The washing step was repeated and the alkaline phosphatase‐conjugated secondary antibody (Cell Signaling, 7054S) was added for 2.5 h. The washing step was repeated, and the bands were developed using a Bio‐Rad alkaline phosphatase development kit according to the manufacturer's instructions.

Densitometry analysis was performed using ImageJ software (version 1.53 t). Protein expression was normalized to total protein, which was determined using densitometry analysis performed on the duplicate gel. The fold change in protein expression was determined relative to the untreated control.

### 2.7 Biolog phenotype microarrays

A 2× assay mix containing mitochondrial assay solution (MAS, Biolog), 2× Redox Dye MC (Biolog), and 100 μg/mL saponin (Sigma‐Aldrich) was prepared. The cannabinoid treatments were prepared in the assay mix, added to the respective wells of the MitoPlate™ S‐1 (Biolog), and incubated at 37°C for 1 h.

Trypsinized cells were collected and centrifuged (36 × *g*) for 5 min, the supernatant removed, and cells resuspended in 1× MAS. Cells were counted and diluted to 1,000,000 cells/mL before being added to each well at a final concentration of 30,000 cells/well. The absorbance (590 and 750 nm) was measured every 5 min for 10 h. The area under the curve between each consecutive point was calculated using the trapezoidal method.

### 2.8 Multicellular tumour spheroids

#### 2.8.1 Spheroid formation

The adherent tissue culture‐treated surface of 96‐well plates was coated with 100 μL agarose (1% m/v). Cells were seeded at a density of 6000 cells/well. The respective treatments were added to the cells and spheroid formation was monitored by acquiring images after Day 1 and 2 of incubation.

#### 2.8.2 Spheroid migration

The adherent tissue culture‐treated surface of 96‐well plates was coated with 100 μL agarose (1% m/v). Cells were seeded at a density of 6000 cells/well and incubated for 10 days. Half the media volume was replaced with fresh media on Days 3 and 6. On Day 10, the spheroids were transferred to a 96‐well plate with the attachment surface exposed (*t* = 0). The treatment was also added at this point and image acquisition was used to monitor spheroid migration. Fresh treatment was added on Day 2.

#### 2.8.3 Spheroid treatment

The adherent tissue culture‐treated surface of 96‐well plates was coated with 100 μL agarose (1% m/v). Cells were seeded at a density of 6000 cells/well and incubated for 10 days. Half the media volume was replaced with fresh media on Days 3 and 6. On Day 10, the spheroids were transferred to a 96‐well plate with agarose coating the attachment surface (*t* = 0). The treatment was also added at this point and the spheroids were monitored by image acquisition. Fresh treatment was added on Day 2.

### 2.9 Statistical analysis

All experiments were performed at least three times (*n* = 3) with at least three repetitions per experiment. Data were plotted using GraphPad Prism and expressed as means ± standard deviation (SD). The Real Statistics Resource Pack software (Release 8.3.1) and copyright (2013–2023) available at www.real-statistics.com ^28^ were used for statistical analysis. Significance was determined using multiple comparisons with a one‐factor analysis of variance (ANOVA) and post‐hoc Tukey test with Bonferroni alpha correction for contrasts. Statistical significance was set at *p* < 0.05.

## 3 RESULTS AND DISCUSSION

### 3.1 The cannabinoid ratio exerted a concentration‐dependent cytotoxic effect on MCF7 cells

MCF7 and MCF10A cell lines were treated with increasing concentrations of the ratio for 24 h, and the effect on cell viability and morphology was determined.

**FIGURE 1:**
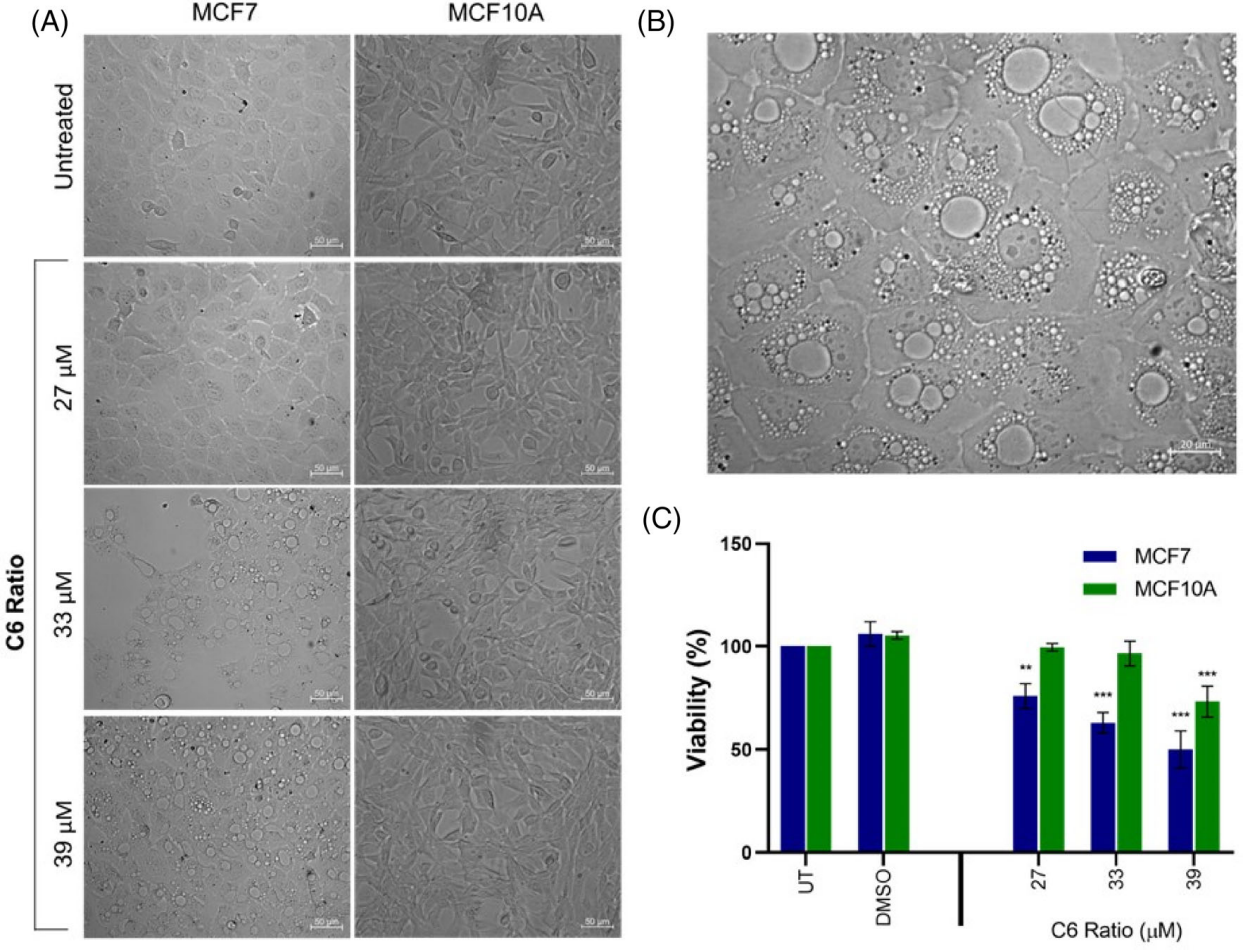
Effect of the cannabinoid ratio on cell viability and morphology. (A) Change in the morphology of the MCF7 and MCF10A cell lines after treatment with increasing concentrations of the cannabinoid ratio for 24 h and (B) the change in morphology of the MCF7 cell line after treatment with 39 μM of the cannabinoid ratio, determined using light microscopy (20× and 40× objective, scale bar = 50 μm and 20 μm). (C) Change in the viability of MCF7 and MCF10A cell lines after treatment with increasing concentrations of the cannabinoid ratio for 24 h, determined using the crystal violet assay (*n* = 3, data are presented as mean ± SD, *significant to untreated control; **p < 0.01; ***p < 0.001). DMSO, dimethyl sulfoxide vehicle control; UT, untreated.

Treatment with the cannabinoid ratio resulted in a concentration‐dependent increase in the formation of cytoplasmic vacuoles in the tumorigenic MCF7 cell line (Figure 1A), particularly at the highest concentration (Figure 1B). No notable morphological changes were induced in the non‐tumorigenic MCF10A cell line. The MCF7 cells also exhibited a significant concentration‐dependent decrease in viability after treatment with the ratio, with no significant decrease observed in the MCF10A cells at the lower cannabinoid concentrations (Figure 1C). This suggested that the cytotoxic mechanism induced by the ratio was more specific to the tumorigenic MCF7 cell line.

### 3.2 The cannabinoid ratio did not induce cell cycle arrest or any common cell death mechanisms

The effect of the ratio on cell cycle distribution, apoptosis, and necrosis was measured using FITC Annexin V, Hoechst, and PI staining. Camptothecin (CPT), a known inducer of G2/M phase arrest and apoptosis, was used as a positive control.^29^ CPT was used to indicate that the method used to detect cell cycle arrest and apoptosis was sound and not as a comparison of the treatment efficacies.

**FIGURE 2:**
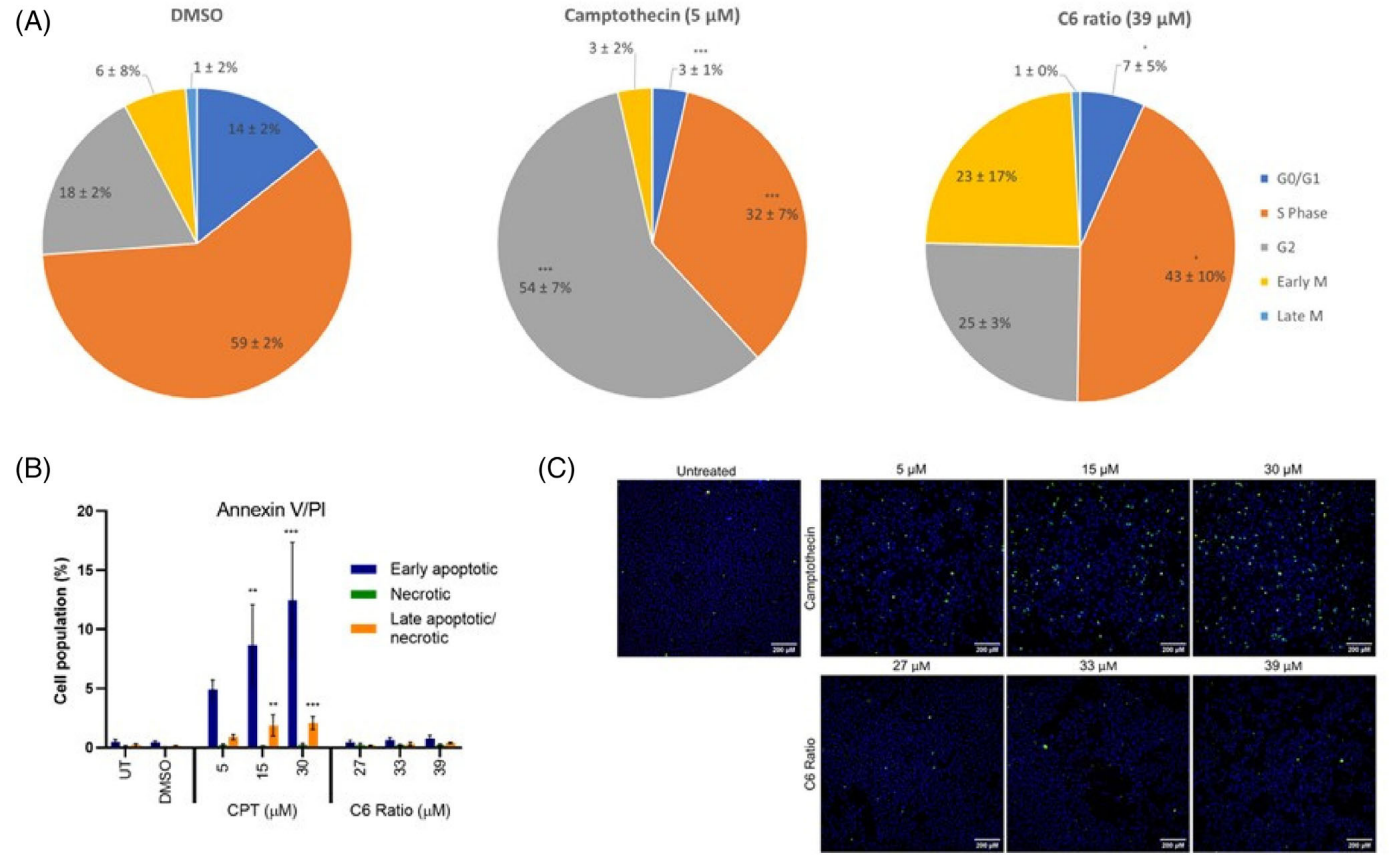
Effect of the cannabinoid ratio on the induction of cell cycle arrest, apoptosis, and necrosis in the MCF7 cell line. (A) Distribution of the MCF7 cell population in the various phases of the cell cycle and (B, C) the induction of apoptosis and necrosis after treatment with camptothecin and the cannabinoid ratio for 24 h, determined using fluorescent staining with Hoechst, FITC Annexin V, and propidium iodide. Image acquisition (10× objective, scale bar = 200 μm) and analysis were completed using the ImageXpress High‐Content Analysis System (*n* = 3, data are presented as mean ± SD, *significant compared to untreated control; *p < 0.05; **p < 0.01; ***p < 0.001). CPT, camptothecin; DMSO, dimethyl sulfoxide vehicle control; UT, untreated.

Cells treated with CPT showed significant G2 phase arrest (Figure 2A) and the concentration‐dependent induction of apoptosis in MCF7 cells (Figure 2B). Cells treated with the cannabinoid ratio showed a slight, non‐significant increase in the cell population in the G2 and M phases (Figure 2A). No increase in the induction of apoptosis or necrosis was observed, with less than 2% of the cell population being recorded as apoptotic or necrotic (Figure 2B,C). Schoeman et al.^23^ found that treatment with the ratio induced significant G2 phase arrest and apoptosis in the MCF7 cells after 48 h, which was not observed after 24 h, suggesting that an alternative mechanism was responsible for the significant cell death observed after 24 h of treatment.

Autophagy was assessed using the autophagy inhibitor 3‐methyladenine (3‐MA), which inhibits class III phosphoinositide 3‐kinase (PI3K) which is crucial for autophagy induction.^30^ The LysoTracker™ Green fluorescent dye, which accumulates in acidic cellular compartments such as lysosomes, was also used to measure autophagy. Tunicamycin (TNC), a known inducer of ER stress and autophagy, was used as a positive control.^31^

**FIGURE 3:**
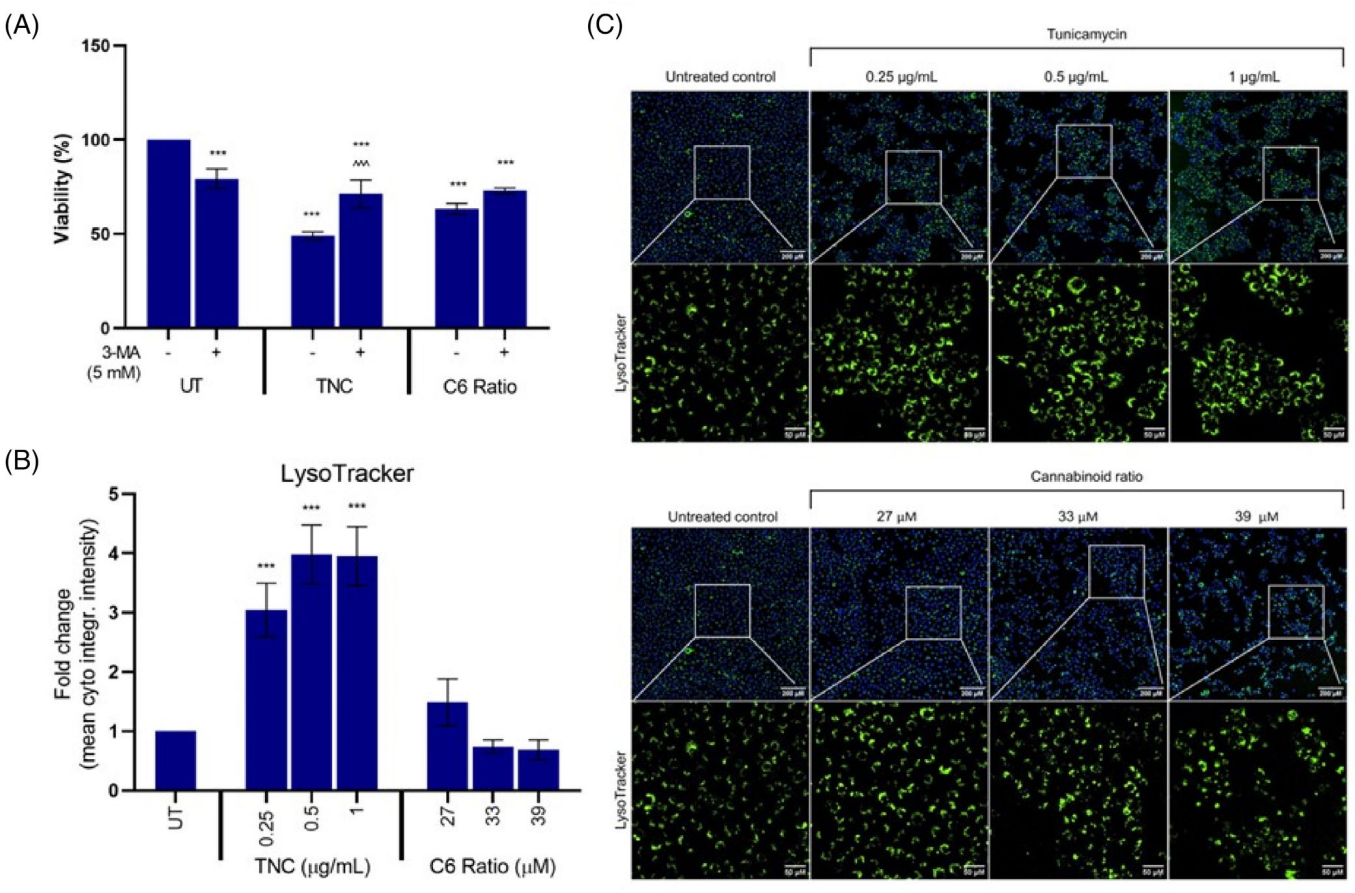
Effect of the cannabinoid ratio on the induction of autophagy in the MCF7 cell line. (A) The effect of autophagy inhibition with 3‐MA on the viability of cells treated with TNC and the cannabinoid ratio for 24 h, were determined using the crystal violet assay. (B, C) The effect of treatment with TNC and the cannabinoid ratio on LysoTracker staining intensity. Image acquisition (10× objective, scale bar = 200 μm and 50 μM) and analysis were completed using the ImageXpress High‐Content Analysis System (*n* = 3, data are presented as mean ± SD, *significant compared to untreated control; ^significant to respective treatment alone, without 3‐MA; ***/^^^*p* < 0.001). 3‐MA, 3‐methyladenine; DMSO, dimethyl sulfoxide vehicle control; TNC, tunicamycin; UT, untreated.

Concomitant treatment with 3‐MA significantly increased the viability of cells treated with TNC (Figure 3A), suggesting effective inhibition of autophagy. However, autophagy inhibition did not affect cell death induced by the cannabinoid ratio, suggesting that autophagy was not induced. Treated cells were stained with LysoTracker™ Green fluorescent dye to test this further. Treatment with TNC significantly increased staining intensity, suggested an increase in the formation of lysosomes (Figure 3B,C); however, no increase in LysoTracker staining intensity was observed after treatment with the cannabinoid ratio, further suggesting that autophagy was not induced.

### 3.3 The cannabinoid ratio did not induce iron‐ or lipid peroxidation‐dependent cell death

Ferroptosis, characterized by the iron‐dependent formation of lipid peroxides,^32^ was also investigated. RAS‐selective lethal 3 (RSL3) was used as a positive control.^32^, ^33^ Cells were concomitantly treated with deferoxamine (DFO, an iron chelator) and ferrostatin‐1 (FER‐1, a lipid peroxidation inhibitor),^32^ and the effect on cell viability was measured to determine whether cell death was dependent on iron and/or lipid peroxidation.

**FIGURE 4:**
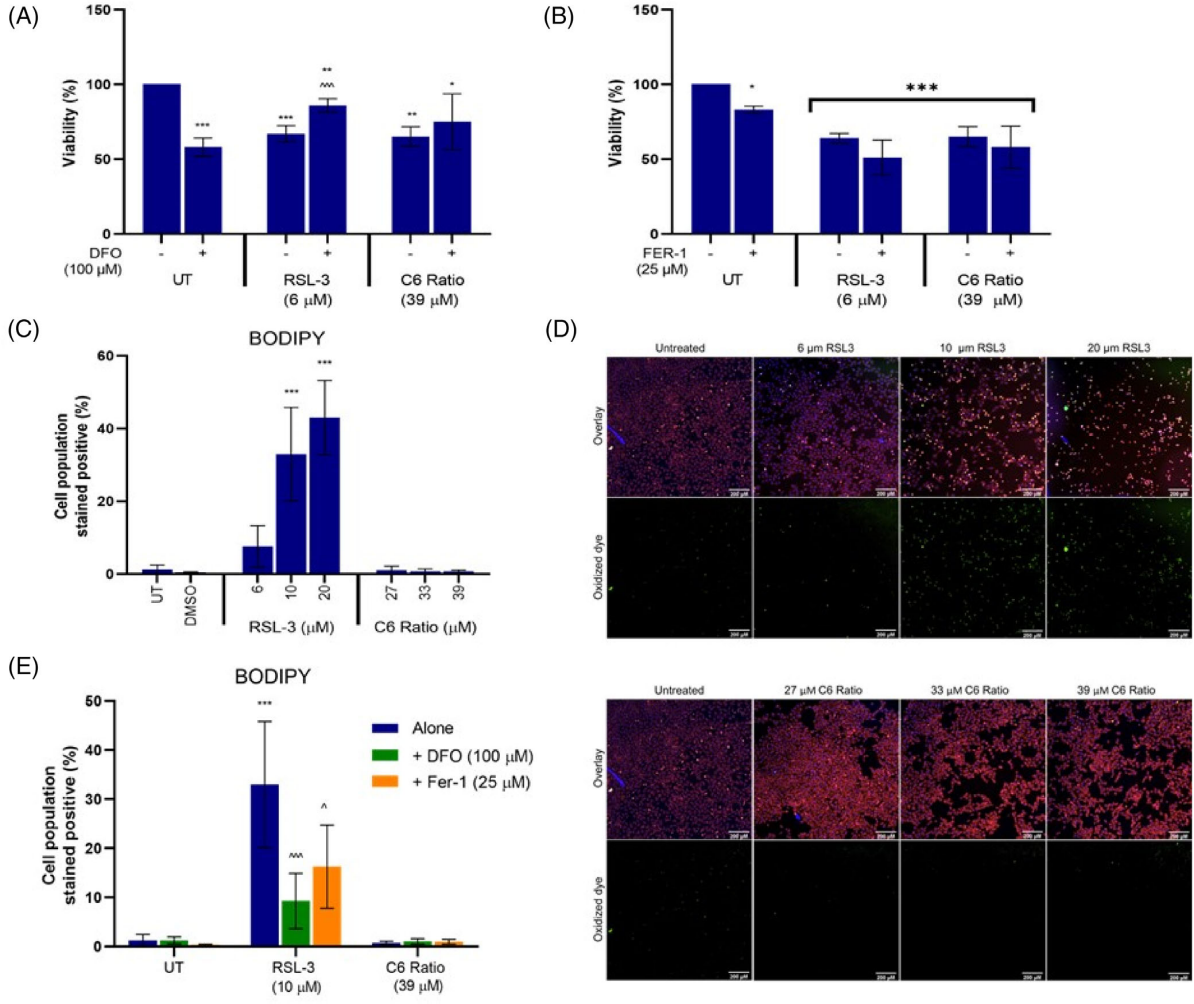
Effect of the cannabinoid ratio on ferroptosis induction in the MCF7 cell line. The effect of concomitant (A) DFO and (B) FER‐1 treatment on the viability of cells treated with RSL3 and the cannabinoid ratio for 24 h, were determined using the crystal violet assay. (C, D) The effect of treatment with RSL3 and the cannabinoid ratio and (E) concomitant treatment with DFO and FER‐1 on lipid peroxidation, determined using C11‐BODIPY™ staining. Image acquisition (10× objective, scale bar = 200 μm) and analysis were completed using the ImageXpress High‐Content Analysis System (*n* = 3, data are presented as mean ± SD, *significant to untreated control; ^significant to respective treatment alone, without DFO or FER‐1; */^*p* < 0.05; **p < 0.01; ***/^^^*p* < 0.001). DFO, deferoxamine; DMSO, dimethyl sulfoxide vehicle control; FER‐1, Ferrostatin‐1; RSL3, Ras‐lethal selective 3; UT, untreated.

Concomitant DFO treatment significantly increased the viability of RSL3‐treated cells, but had no significant effect on cannabinoid‐treated cells (Figure 4A). This suggested that cell death induced by the ratio was not significantly iron‐dependent.

Concomitant treatment with FER‐1 had no effect on the viability of RSL3‐ or cannabinoid‐treated cells (Figure 4B); therefore, BODIPY™ C11, a fluorescent reporter of lipid peroxidation, was used to directly test for the induction of lipid peroxidation. Upon oxidation, BODIPY™ fluorescence shifts from red to green. RSL3 treatment resulted in a concentration‐dependent increase in the population of cells that exhibited green fluorescence (Figure 4C,D), suggesting an increase in lipid peroxidation. In contrast, cells treated with the cannabinoid ratio showed no increase in green fluorescence, with less than 1% of the cell population staining positive for the oxidized dye. Concomitant treatment with DFO and FER‐1 significantly decreased lipid peroxidation in RSL3‐treated cells (Figure 4E), confirming that RSL3 induced iron‐dependent lipid peroxidation. However, concomitant treatment did not affect lipid peroxidation in cannabinoid‐treated cells, further confirming that ferroptosis was not induced.

### 3.4 The cannabinoid ratio induces paraptosis‐like cell death

Paraptosis was investigated as a potential mechanism of cell death. Morphological analysis using light microscopy showed that the cannabinoid ratio induced significant vacuolation (Figure 1A,B; Video S1), which was further investigated through transmission electron microscopy (TEM). Another characteristic of paraptosis is a dependence on protein translation, as one of the mechanisms of paraptosis induction is impaired proteostasis.^20^, ^21^ Therefore, paraptosis induction is often confirmed by inhibiting protein translation using cycloheximide (CHX).^18^ TNC was used as a positive control, due to its dependence on protein translation to exert its effect as an ER stress inducer. It has also been shown to induce paraptosis independent of ER stress induction.^34^

**FIGURE 5:**
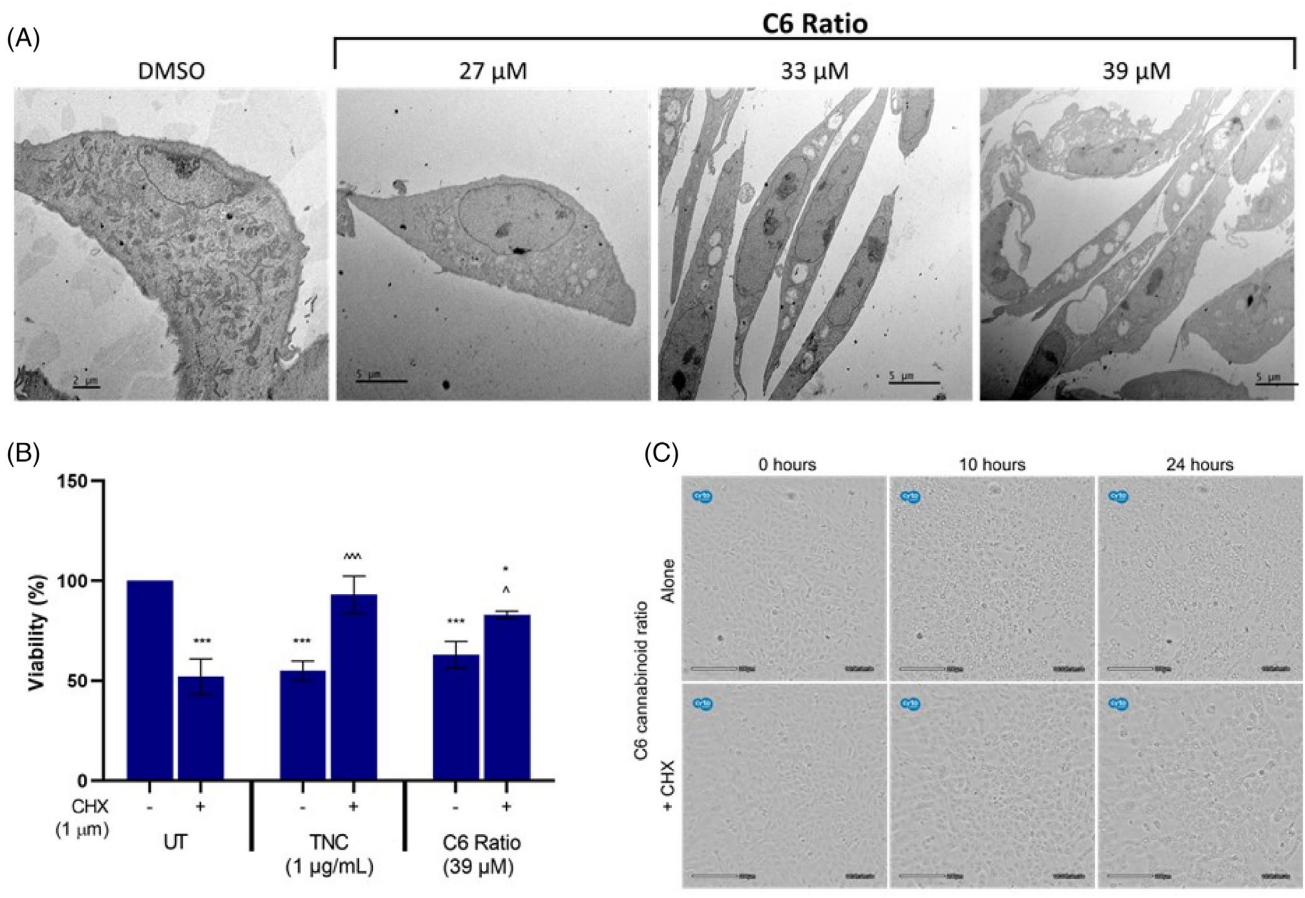
The effect of the cannabinoid ratio on paraptosis induction in the MCF7 cell line. (A) Representative TEM micrographs showing the change in morphology of cells treated with the cannabinoid ratio for 24 h. (B) The effect of concomitant treatment with CHX on the viability of cells treated with TNC and the cannabinoid ratio, was determined using crystal violet staining. (C) The effect of concomitant CHX treatment on the morphology of cells treated with the cannabinoid ratio (39 μM), monitored using the CytoSMART Lux2 live‐cell imager (*n* = 3, data are presented as mean ± SD, *significant to untreated control; ^significant to respective treatment alone, without CHX; */^*p* < 0.05; ***/^^^*p* < 0.001). CHX, cycloheximide; DMSO, dimethyl sulfoxide vehicle control; TNC, tunicamycin; UT, untreated.

TEM analysis showed a concentration‐dependent induction of vacuoles in the MCF7 cells (Figure 5A), which is characteristic of paraptosis. Concomitant CHX treatment significantly increased the viability of both TNC‐ and cannabinoid‐treated cells (Figure 5B), and markedly suppressed cytoplasmic vacuolation induced by the cannabinoid ratio (Figure 5C; Video S2). This suggested that the vacuolation and cell death induced by the ratio may, at least partially, depend on protein translation.

These results suggested that paraptosis was induced by the cannabinoid ratio. Paraptosis is often associated with the induction of ER stress, alteration of redox homeostasis, mitochondrial swelling, and a disruption of Ca^2+^ signalling,^20^ which were investigated.

### 3.5 Paraptosis induced by the cannabinoid ratio is independent of ROS generation

Cells were concomitantly treated with two antioxidants, ascorbic acid (AA) and N‐acetylcysteine (NAC) to determine the effect of the cannabinoid ratio on ROS production.

**FIGURE 6:**
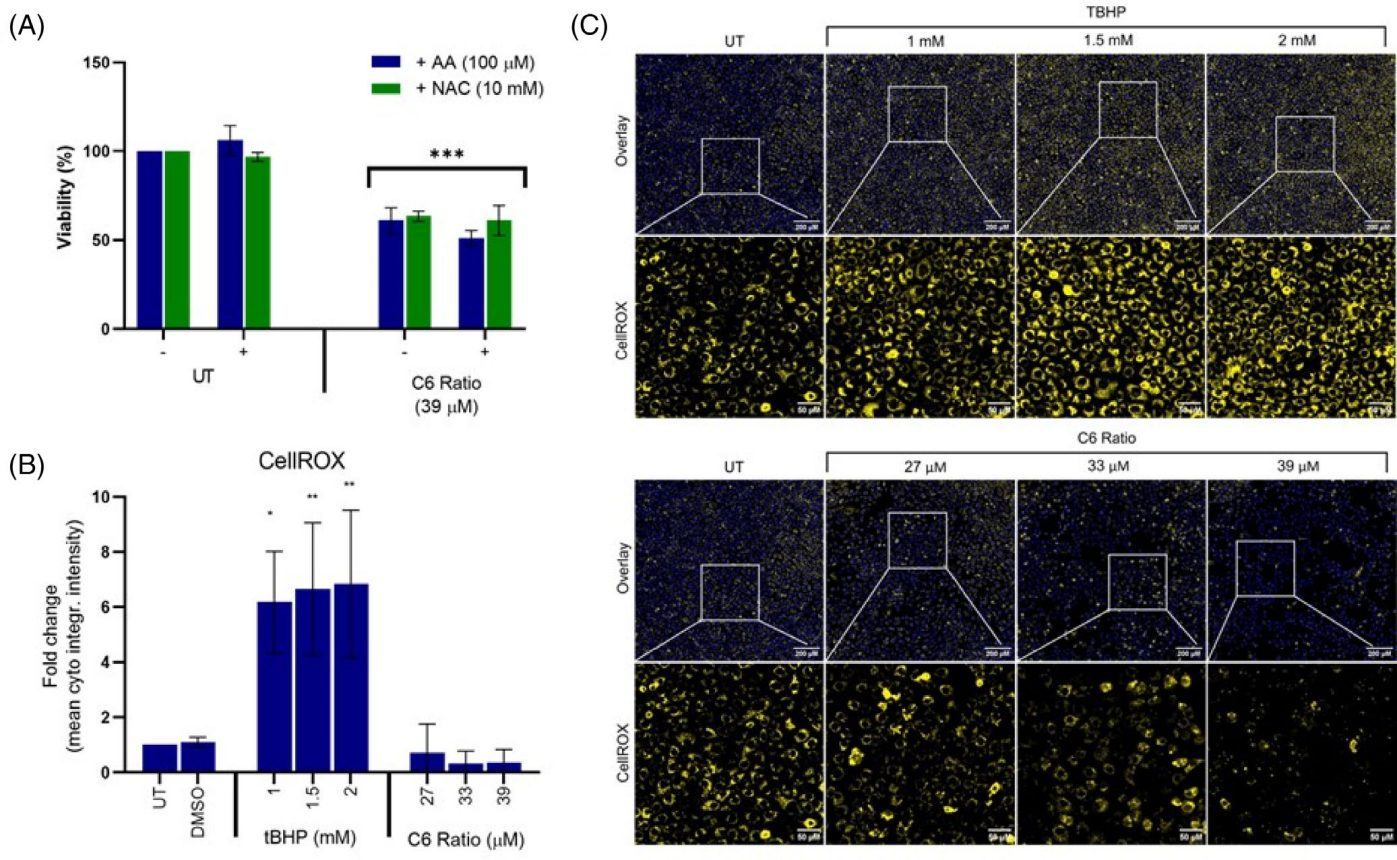
The effect of the cannabinoid ratio on ROS generation in the MCF7 cell line. (A) The effect of concomitant treatment with AA and NAC on the viability of cells treated with the cannabinoid ratio, was determined using the crystal violet assay. (B, C) The effect of treatment with tBHP and the cannabinoid ratio on ROS generation, was determined using CellROX™ Orange staining. Image acquisition (10× objective, scale bar = 200 μM and 50 μM) and analysis were completed using the ImageXpress High‐Content Analysis System (*n* = 3, data are presented as mean ± SD, *significant to untreated control; *p < 0.05; **p < 0.01; ***p < 0.001). AA, ascorbic acid; DMSO, dimethyl sulfoxide vehicle control; NAC, N‐acetyl cysteine; TBHP, tert‐butyl hydroperoxide; UT, untreated.

Neither AA nor NAC had any effect on cell viability (Figure 6A), suggesting that ROS production did not play a role in cell death and vacuolation induced by the ratio. The CellROX™ orange stain for oxidative stress confirmed this, with tert‐butyl hydroperoxide (tBHP) being used as a positive control. A significant increase in the CellROX™ staining intensity was observed after treatment with tBHP (Figure 6B,C), with no increase in staining intensity observed after cannabinoid treatment, confirming that ROS generation was not increased and did not appear to play a role in paraptosis induced by the cannabinoid ratio.

### 3.6 The cannabinoid ratio induces significant ER stress

Western blot analysis for glucose‐regulated protein 78 (GRP78) and C/EBP homologous protein (CHOP) expression was used to detect ER stress, as they are both upregulated by the unfolded protein response (UPR) during periods of ER stress.^35^, ^36^ TNC, a known ER stress inducer, was used as a positive control.

**FIGURE 7:**
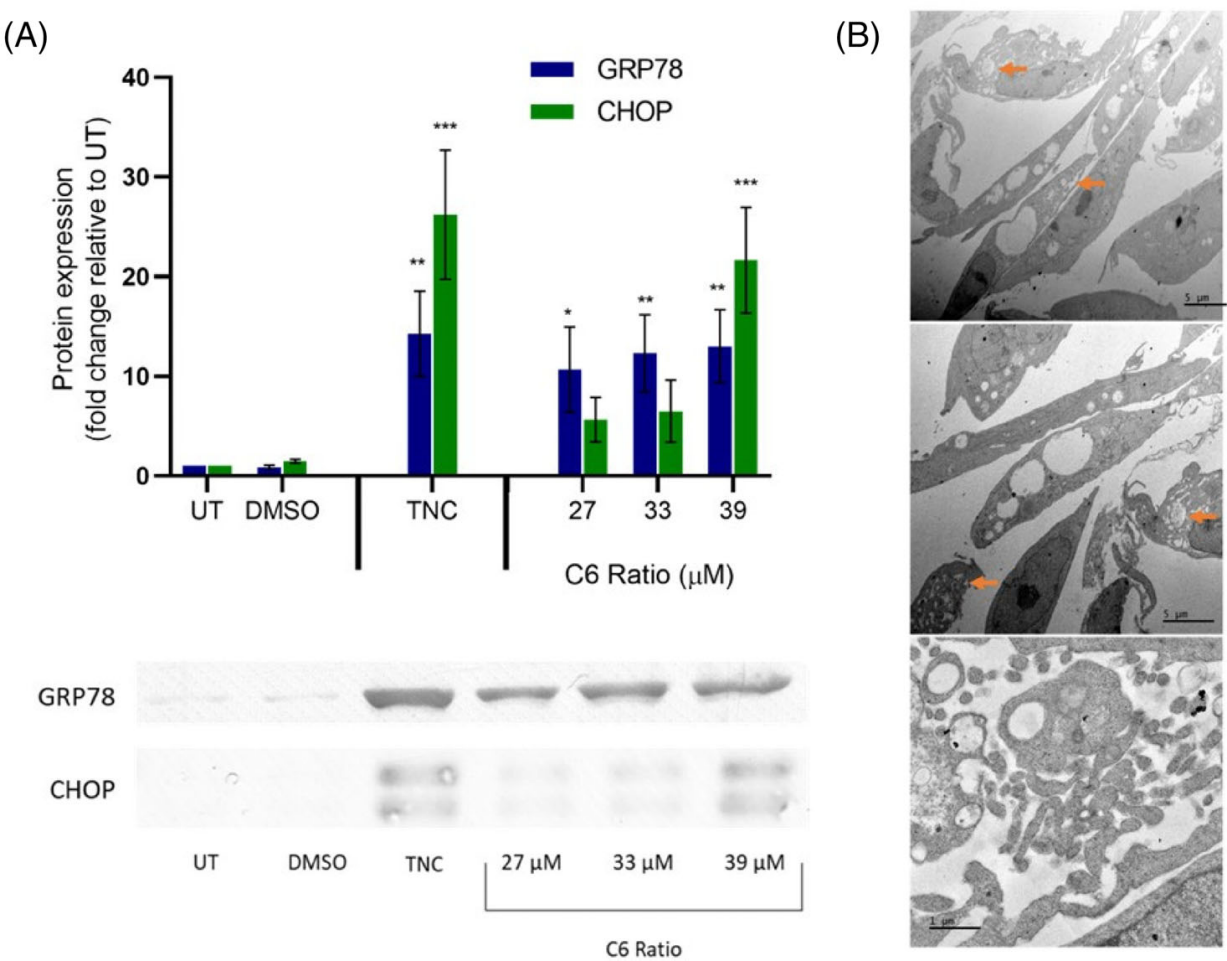
The effect of the cannabinoid ratio on ER stress induction in the MCF7 cell line. (A) The effect of treatment with TNC (1 μg/mL) and the cannabinoid ratio for 24 h on GRP78 and CHOP expression, was determined using Western blot analysis. (B) Representative TEM micrographs showing the effect of treatment with the cannabinoid ratio (39 μM) on ER dilation (orange arrows) (*n* = 3, data are presented as mean ± SD, *significant to untreated control; *p < 0.05; **p < 0.01; ***p < 0.001). DMSO, dimethyl sulfoxide vehicle control; GRP78, glucose‐regulated protein; TNC, tunicamycin; UT, untreated.

Both TNC and the cannabinoid ratio significantly increased GRP78 expression (Figure 7A). Treatment with TNC and the highest concentration of the cannabinoid ratio significantly increased CHOP expression. CHOP was detected as a doublet, which may indicate stress‐induced phosphorylation by stress‐inducible members of the P38 MAPK family.^37^, ^38^ The significant increase in GRP78 and CHOP expression observed after treatment with the cannabinoid ratio suggested significant ER stress induction and UPR activation. TEM analysis also showed that cells treated with the highest concentration of the cannabinoid ratio (39 μM) had swollen ER cisternae (Figure 7B, orange arrows), further indicating severe ER stress induction.

### 3.7 The cannabinoid ratio induces significant changes to mitochondrial structure and function

The effect of the cannabinoid ratio on mitochondrial structure and function was determined using TEM analysis, CytoPainter Orange, and nonyl acridine orange (NAO) staining.

**FIGURE 8:**
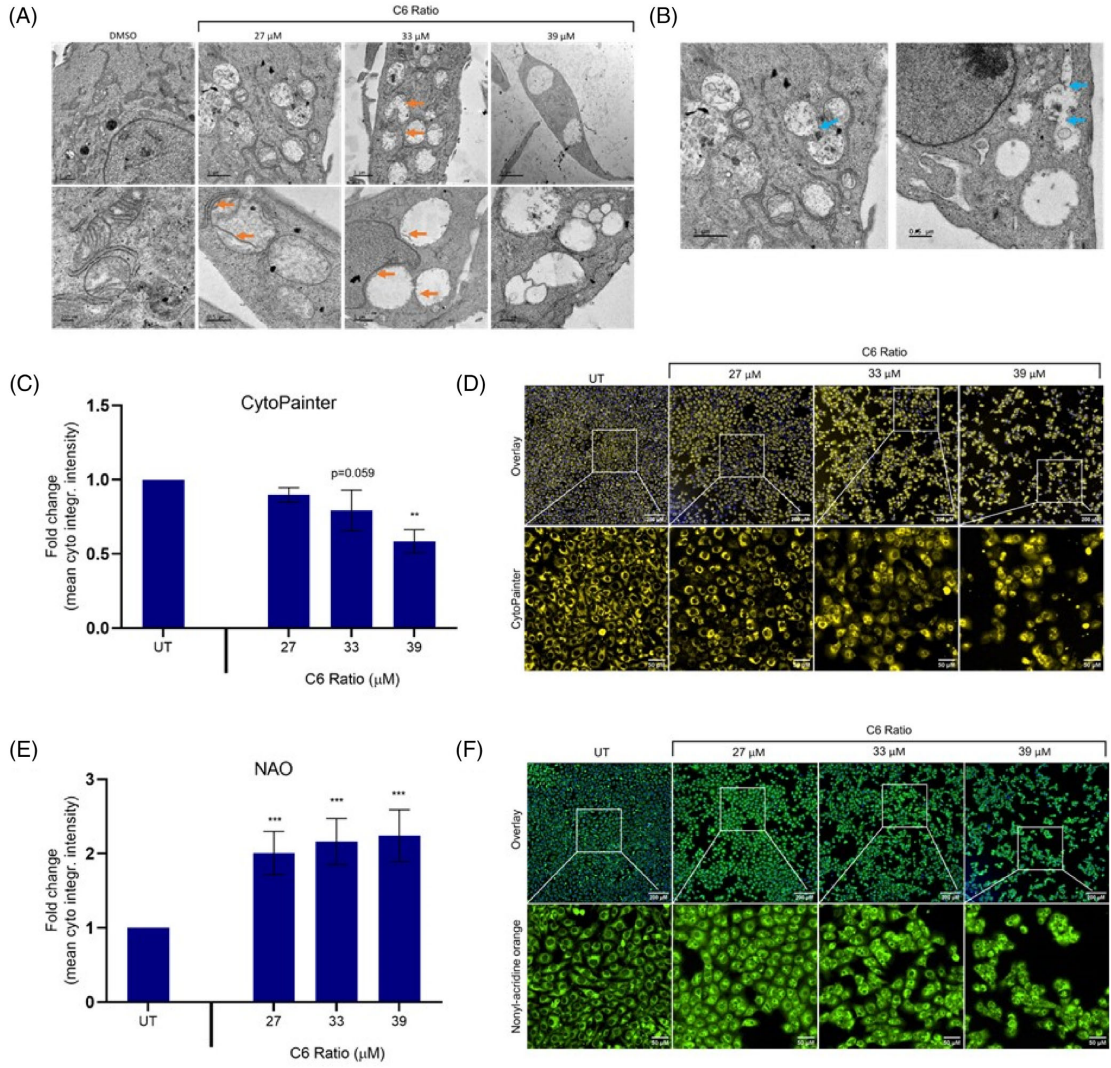
The effect of the cannabinoid ratio on mitochondrial structure and function in the MCF7 cell line. (A) Representative TEM micrographs showing the effect of treatment with the cannabinoid ratio for 24 h on mitochondrial structure and (B) mitochondrial fusion. (C, D) The effect of treatment with the cannabinoid ratio on the mitochondrial membrane potential and (E, F) mitochondrial mass, was determined using CytoPainter Orange and NAO staining, respectively. Image acquisition (10× objective, scale bar = 200 μM and 50 μM) and analysis were completed using the ImageXpress High‐Content Analysis System (*n* = 3, data are presented as mean ± SD, *significant to untreated control; **p < 0.01; ***p < 0.001). DMSO, dimethyl sulfoxide vehicle control; NAO, nonyl acridine orange; UT, untreated.

TEM analysis showed that the cannabinoid ratio induced significant mitochondrial swelling and disruption to the mitochondrial structure (Figure 8A). The remnants of the mitochondrial cristae can be seen at the edges of the swollen mitochondria (Figure 8A, orange arrows). Mitochondrial swelling as well as disintegration and loss of the mitochondrial cristae are typical characteristics of paraptosis,^20^ both of which were observed. Many natural products that induce paraptotic cell death cause mitochondrial swelling and fusion, leading to the formation of megamitochondria structures.^20^, ^39^, ^40^, ^41^, ^42^ This appears to have been induced by the cannabinoid ratio, as the TEM micrographs show areas that suggest mitochondrial fusion (Figure 8B, blue arrows) which, along with mitochondrial swelling, may explain the large megamitochondria structures observed.

The CytoPainter fluorescent stain was used to measure changes in the mitochondrial membrane potential (MMP). Staining and analysis showed that the cannabinoid ratio induced a significant, concentration‐dependent decrease in the MMP (Figure 8C,D), indicating that mitochondrial dysfunction was induced. The cannabinoid ratio also induced a significant increase in NAO staining (Figure 8E,F), which binds to cardiolipin (CL) in the inner mitochondrial membrane and is generally used to investigate changes in mitochondrial mass. CL is central to many mitochondrial processes, including: maintaining mitochondrial cristae morphology and stability, mitochondrial quality control, mitochondrial dynamics through fission and fusion, mitochondrial biogenesis, and mitophagy.^43^ The significant increase in CL may have been induced to stabilize the mitochondrial function and/or cristae morphology, to induce mitophagy to remove damaged mitochondria, or to induce mitochondrial biogenesis and/or fusion in response to compromised mitochondrial function.

### 3.8 Paraptosis induced by the cannabinoid ratio was primarily dependent on dysregulated calcium signalling

Many plant extracts that induce paraptosis have been shown to mediate Ca^2+^ flux from the ER to the mitochondria, primarily through the voltage‐dependent anion channel (VDAC).^44^ Cells were concomitantly treated with 4,4′‐Diisothiocyanatostilbene‐2,2′‐disulfonate (DIDS), a VDAC inhibitor,^45^, ^46^ to investigate the effect of the cannabinoid ratio on Ca^2+^ dysregulation. Treated cells were also stained with Rhod‐2 AM, a fluorescent Ca^2+^ stain that accumulates in the mitochondria and was used to monitor mitochondrial Ca^2+^ levels.^47^

**FIGURE 9:**
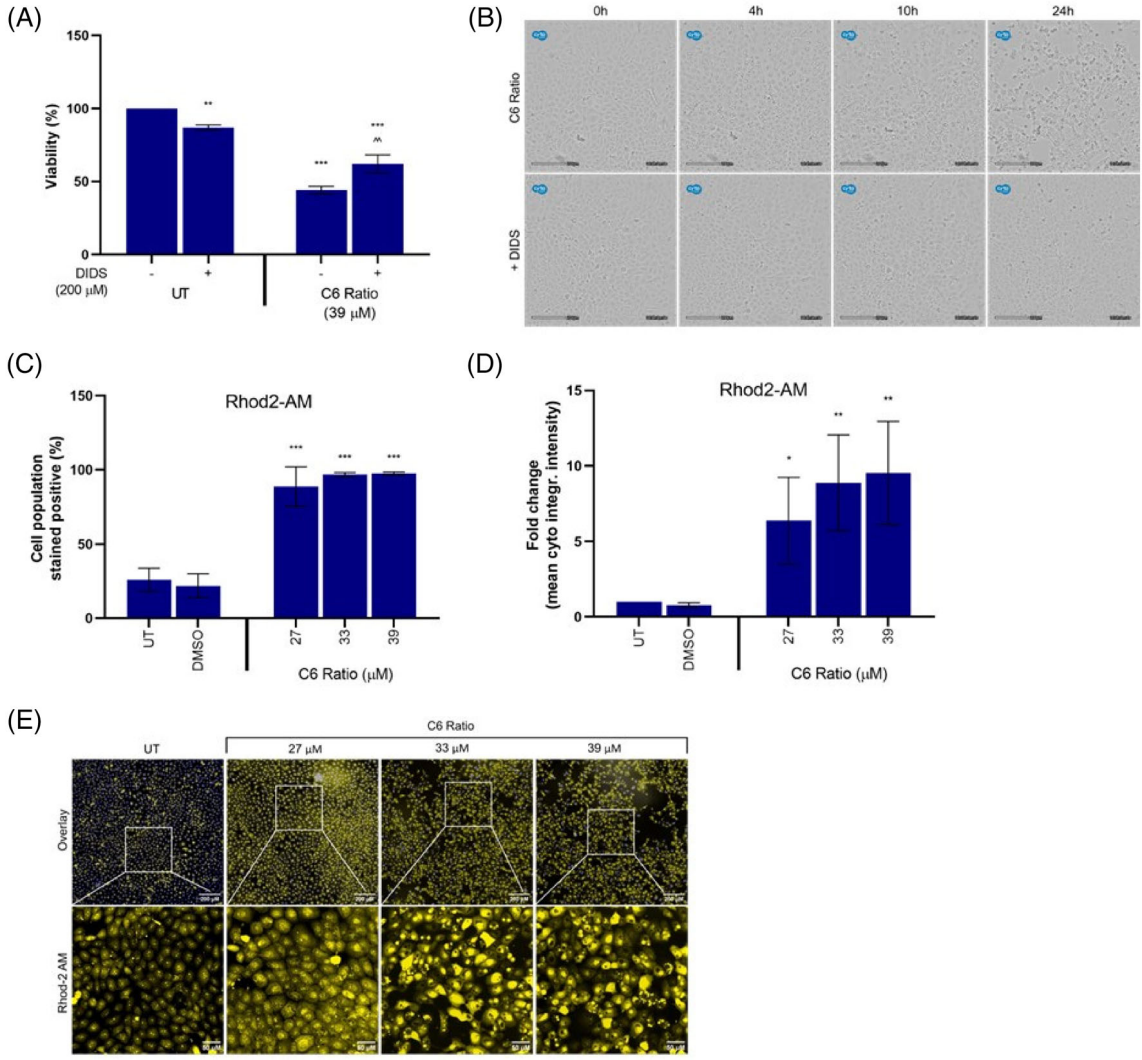
The effect of the cannabinoid ratio on calcium dysregulation in the MCF7 cell line. The effect of concomitant DIDS treatment on the (A) viability and (B) morphology of cells treated with the cannabinoid ratio for 24 h, was determined using crystal violet staining and the CytoSMART Lux2 live‐cell imager, respectively. (C–E) The effect of treatment with the cannabinoid ratio on mitochondrial calcium accumulation, was determined using Rhod‐2 AM staining. Image acquisition (10× objective scale bar = 200 μM and 50 μM) and analysis were completed using the ImageXpress High‐Content Analysis System (*n* = 3, data are presented as mean ± SD, *significant to untreated control, ^significant to respective treatment alone, without DIDS; *p < 0.05; **p < 0.01; ***p < 0.001). DIDS, 4,4′‐Diisothiocyanatostilbene‐2,2′‐disulfonate; DMSO, dimethyl sulfoxide vehicle control; UT, untreated.

Concomitant treatment with DIDS significantly increased viability (Figure 9A) and inhibited vacuole formation in cannabinoid‐treated cells (Figure 9B; Video S3), suggesting that many of the effects observed after cannabinoid treatment may be due to Ca^2+^ influx into the mitochondria. A significant increase in the population of cells that stained positive for Rhod‐2 AM (Figure 9C), as well as a significant, concentration‐dependent increase in staining intensity (Figure 9D,E), was observed. This suggested that the cannabinoid ratio induced a significant increase in mitochondrial Ca^2+^ levels.

Increased Ca^2+^ flux into the mitochondria explains many effects observed after treatment with the cannabinoid ratio, including mitochondrial dilation and loss of structure, decreased MMP, ER dilation, and ER stress. The mitochondrial matrix volume is controlled by an osmotic balance between the cytosol and mitochondrial matrix.^48^ An imbalance of Ca^2+^ and K^+^ ions between the cytosol and mitochondrial matrix increases osmotic pressure and causes water influx into the matrix, leading to swelling.^48^, ^49^ Calcium overload in the mitochondrial matrix also leads to the opening of the mitochondrial permeability transition pore (mPTP) causing an increase in the permeability of the inner mitochondrial membrane and dissipation of the MMP.^50^, ^51^, ^52^

The simultaneous dilation of the ER and mitochondria, as well as the induction of ER stress suggested an interconnected mechanism between these two organelles. Calcium import into the ER is regulated by the sarcoplasmic/endoplasmic reticulum calcium ATPase (SERCA) pumps and is released from ER stores via inositol 1,4,5,‐triphosphate receptors (IP3Rs) and ryanodine receptors (RyRs).^53^, ^54^ IP3Rs and RyRs are generally clustered in areas known as mitochondrial‐associated membranes (MAMs) where the membrane and luminal components of these two organelles can intermix and exchange.^55^ Ca^2+^ released from IP3Rs and RyRs enter the mitochondrial intermembrane space via VDAC before entering the mitochondrial matrix via the mitochondrial Ca^2+^ uniporter (MCU).^56^, ^57^, ^58^, ^59^ The significant increase in cell viability and suppression of vacuolation observed after VDAC inhibition suggested that the Ca^2+^ influx induced by the cannabinoid ratio could largely be attributed to VDAC modulation. This suggested that the mitochondrial Ca^2+^ overload was mediated by increased Ca^2+^ flux from the ER to the mitochondria. In turn, this would deplete Ca^2+^ stores in the ER leading to ER stress and dilation due to incorrect or insufficient protein folding,^44^ both of which were observed after cannabinoid treatment. Maintenance of sufficient Ca^2+^ levels in the ER is critical for protein folding, as the molecular chaperones that assist with protein folding, such as GRP78, depend on Ca^2+^ for their activity.

Overall, the mechanism being induced by the cannabinoid ratio appeared to be consistent with a model that was proposed by Kim et al.^44^ regarding Ca^2+^‐mediated communication between the ER and mitochondria during paraptosis. They proposed that Ca^2+^ release from the ER and uptake into the mitochondria via VDAC would lead to mitochondrial Ca^2+^ overload and trigger mPTP opening, MMP loss, water uptake into the mitochondrial matrix, and subsequent mitochondrial swelling. Ca^2+^ depletion in the ER would lead to a loss in the activity of Ca^2+^‐dependent chaperones, the accumulation of misfolded proteins, and the induction of ER stress, which leads to ER swelling. This mechanism of paraptosis induction is induced by various natural products such as celastrol,^41^ curcumin,^40^ hesperidin^60^, and morusin^61^ and is consistent with all of the effects induced by the cannabinoid ratio.

Regarding the modulation of VDAC, CBD has been shown to bind to VDAC1 and decrease channel conductance.^62^, ^63^ VDAC1 is less permeable to metabolites in this closed, low‐conductance state, but exhibits increased Ca^2+^ permeability.^64^ Olivas‐Aguirre et al.^63^ showed that CBD directly targeted mitochondria and caused mitochondrial Ca^2+^ overload and swelling, and mPTP opening in acute lymphoblastic leukemia cells. The cannabinoid ratio contained four phytocannabinoids as Schoeman et al.^23^ outlined, namely THC, CBG, CBN, and CBD. MCF7 cells were treated with the cannabinoid ratio, and the ratio with each cannabinoid was independently removed to investigate the role those individual cannabinoids contributed. The cells were also treated with individual cannabinoids at the same concentration as in the cannabinoid ratio.

**FIGURE 10:**
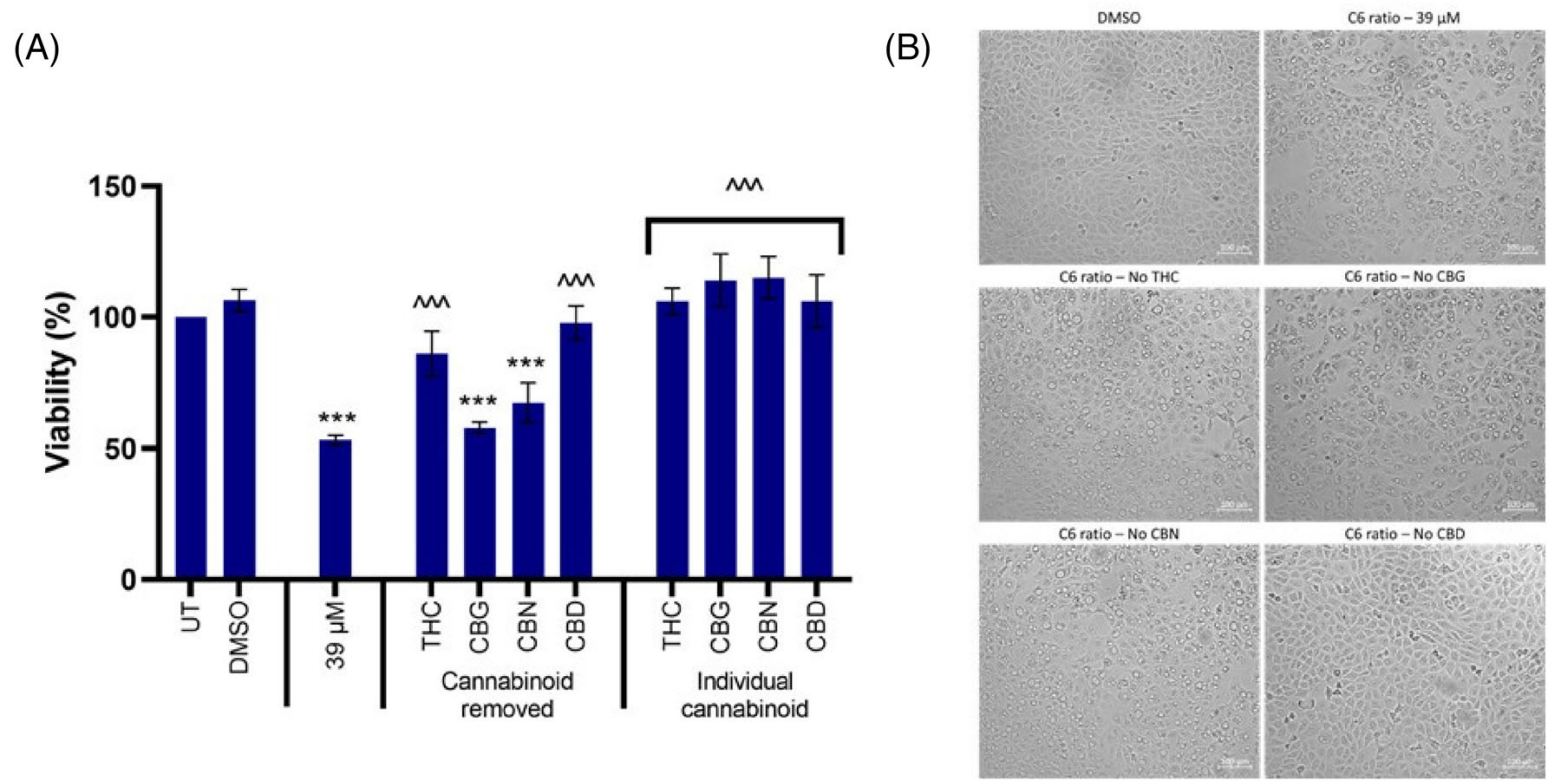
The effect of individual cannabinoids on the effects induced by the cannabinoid ratio in the MCF7 cell line. The effect treatment with the complete cannabinoid ratio, the ratio with individual cannabinoids removed, and individual cannabinoids alone for 24 h on cell (A) viability and (B) morphology, determined using the crystal violet assay and light microscopy (100×, scale bar = 100 μm), respectively (*n* = 3, data are presented as mean ± SD, *significant to untreated control; ^significant to cannabinoid ratio (39 μM);  ***/^^^*p* < 0.001). CBD, Cannabidiol; CBG, Cannabigerol; CBN, Cannabinol; DMSO, dimethyl sulfoxide vehicle control; THC, Δ^9^‐tetrahydrocannabinol; UT, untreated.

Treatment with the complete cannabinoid ratio significantly decreased cell viability and induced extensive vacuolation. The independent removal of CBG and CBN from the ratio had little to no effect on viability (Figure 10A) or vacuolation (Figure 10B). The removal of THC significantly increased viability but had a negligible effect on vacuolation. However, the removal of CBD significantly increased cell viability (Figure 10A), and appeared to completely inhibit vacuolation (Figure 10B), indicating that CBD played a critical role in the vacuolation and cell death induced by the cannabinoid ratio. This data, combined with the studies that have shown that CBD directly binds and modulates VDAC to increase its Ca^2+^ permeability, further supported our theory that VDAC modulation by the cannabinoid ratio is responsible for the dysregulation of Ca^2+^ homeostasis.

Despite the data indicating that CBD is crucial to the effects induced by the cannabinoid ratio, the data also showed that CBD treatment alone—at the same concentration at which it is present in the cannabinoid ratio—had no effect on the viability (Figure 10A) or morphology (data not shown) of the MCF7 cells. This suggested that, while CBD is the most critical cannabinoid in the ratio to induce vacuolation and cell death, it required the presence of the other cannabinoids to work synergistically to exert these effects. Further studies are required to elucidate the exact mechanism by which CBD exhibits an enhanced response in the presence of other cannabinoids.

The characterization of the mechanism of cell death induced by the cannabinoid ratio expands the potential for cannabinoids to be used in the treatment of cancer, particularly in drug‐resistant cancer. Resistance to apoptosis is commonly accepted as a characteristic of cancer cells, and the efficacy of many treatments depends on the cell's susceptibility to apoptosis induction.^65^, ^66^ Therefore, the induction of alternative cell death mechanisms is necessary to treat apoptosis‐resistant cancer. This study highlighted the potential for phytocannabinoids to induce an alternative, non‐apoptotic mechanism of cell death which is essential to overcome drug resistance.

### 3.9 The cannabinoid ratio significantly inhibits mitochondrial metabolism

Changes in mitochondrial metabolism after treatment were measured using the Biolog MitoPlate™ S‐1 platform which measures mitochondrial function by measuring the rate of electron flow through the electron transport chain (ETC) in response to different substrates. The heatmap was plotted using Heatmapper (www.heatmapper.ca)^67^ and the substrates were grouped according to their respective metabolic pathways.^68^

**FIGURE 11:**
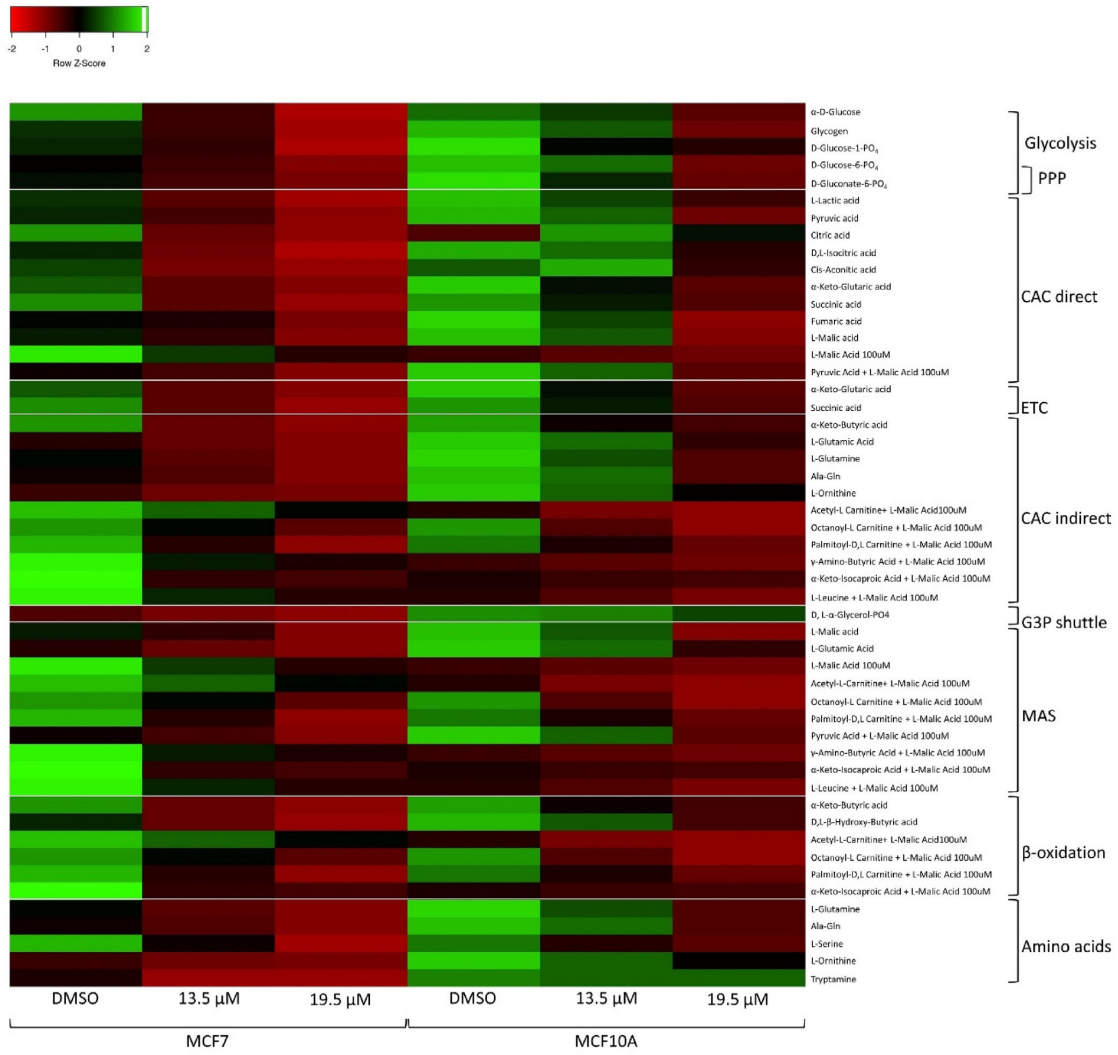
The effect of the cannabinoid ratio on the processing of bioenergetic substrates in the MCF7 cell line. The effect of treatment with DMSO vehicle control, and two concentrations of the cannabinoid ratio (13.5 and 19.5 μM) on the processing of 31 substrates, was determined using the Biolog MitoPlate S‐1. The total area under the curve was calculated and expressed as a fold change relative to the no substrate control of the DMSO vehicle control and plotted as a heat map using Heatmapper with row scaling to compare the treatments (*n* = 3). CAC, citric acid cycle; DMSO, dimethyl sulfoxide vehicle control; G3P, glycerol 3 phosphate; MAS, malate aspartate shuttle; PPP, pentose phosphate pathway.

Cannabinoid treatment inhibited most of the mitochondrial pathways tested, and inhibition appeared to be greater in the MCF7 cell line compared to the MCF10A cell line (Figure 11). The fold change in the area under the curve of the kinetic curves generated for the metabolism of mitochondrial pathways was plotted.

**FIGURE 12:**
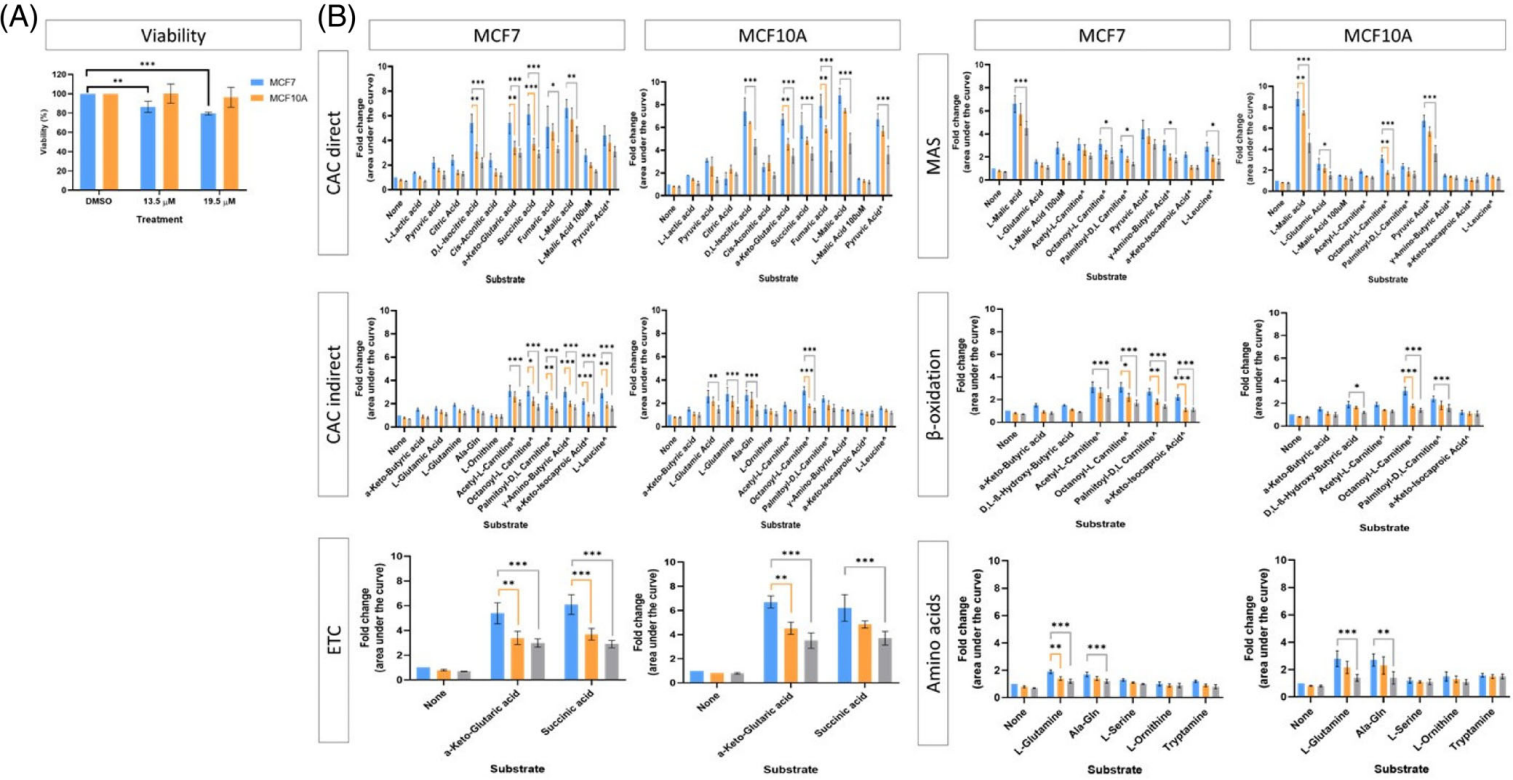
The effect of the cannabinoid ratio on the processing of bioenergetic substrates in the MCF7 cell line. The effect of treatment with the DMSO vehicle control (blue), and two concentrations of the cannabinoid ratio, 13.5 μM (orange), and 19.5 μM (grey) on (A) cell viability, determined using the crystal violet assay, and (B) the processing of 31 substrates, determined using the Biolog MitoPlate S‐1. The total area under the curve was calculated and expressed as a fold change relative to the no substrate control of the DMSO vehicle control (^indicates the substrate is in combination with 100 μM L‐malic acid; *n* = 3, data are presented as mean ± SD, *significant compared to respective DMSO control; *p < 0.05; **p < 0.01; ***p < 0.001). CAC, citric acid cycle; DMSO, dimethyl sulfoxide vehicle control; G3P, glycerol 3 phosphate; MAS, malate aspartate shuttle; PPP, pentose phosphate pathway.

Overall, both cell lines showed inhibition in many of the mitochondrial substrates (Figure 12B); however, the inhibition was generally more significant in the MCF7 cell line compared to the MCF10A cell line. The most significant inhibitions were observed for the direct and indirect substrates of the citric acid cycle (CAC). Most of the substrates that were affected in the MCF7 cells were significantly inhibited at both cannabinoid concentrations, whereas the MCF10A generally only showed significant inhibition at the highest cannabinoid concentration. The inhibition induced in the MCF7 cell line was more general, where most of the mitochondrial pathways tested were significantly inhibited. The inhibition observed in the MCF10A cell line was more specific: significant inhibition was observed in the direct CAC substrates; however, indirect CAC substrates and β‐oxidation substrates were less affected. Despite the significant decreases in metabolism observed in both cell lines, only the MCF7 cell line showed a significant decrease in viability at the concentrations tested after 24 h (Figure 12A). This suggested that the metabolic inhibition induced in the MCF10A cell line by the cannabinoid ratio does not affect the viability of the cells at the concentrations tested, further supporting the selective cytotoxicity of the cannabinoid ratio for cancerous cells compared to non‐cancerous cells.

Increasing evidence is showing that dysregulated bioenergetic metabolism is associated with drug resistance in cancer treatment, and numerous metabolic pathways including glycolysis, CAC, and fatty acid synthesis have been targeted with metabolic inhibitors.^69^ However, the development of drugs that inhibit metabolism has been limited due to their high toxicity to normal cells. Another limitation of the use of metabolic inhibitors is that they generally have extremely high specificity, and the metabolic plasticity of cancer cells enables them to rewire metabolic pathways, which poses a significant challenge to these therapies. Therefore, therapies that block multiple pathways may be more beneficial.^70^, ^71^ The significant inhibition of the bioenergetic pathways induced by the cannabinoid ratio in the MCF7 cell line highlights the potential of cannabinoids to inhibit multiple metabolic pathways via an indirect mechanism that is induced more significantly in tumorigenic cells compared to non‐tumorigenic cells. This approach allows for the inhibition of cancer cell metabolism without the obstacles that are generally faced when using specific metabolic inhibitors namely, toxicity in non‐tumorigenic cells and the metabolic rewiring in cancer cells that leads to treatment resistance.

### 3.10 The cannabinoid ratio decreases the formation, migration, and size of multicellular tumour spheroids

The MCF7 cells were seeded in 96 well plates with the adherent surface coated in agarose to allow the formation of multicellular tumour spheroids (MCTS). The effect of treatment with the cannabinoid ratio on MCTS formation, migration, and size was determined. Tamoxifen, a standard endocrine treatment used to treat hormone receptor‐positive breast cancer, was used as a control.

**FIGURE 13:**
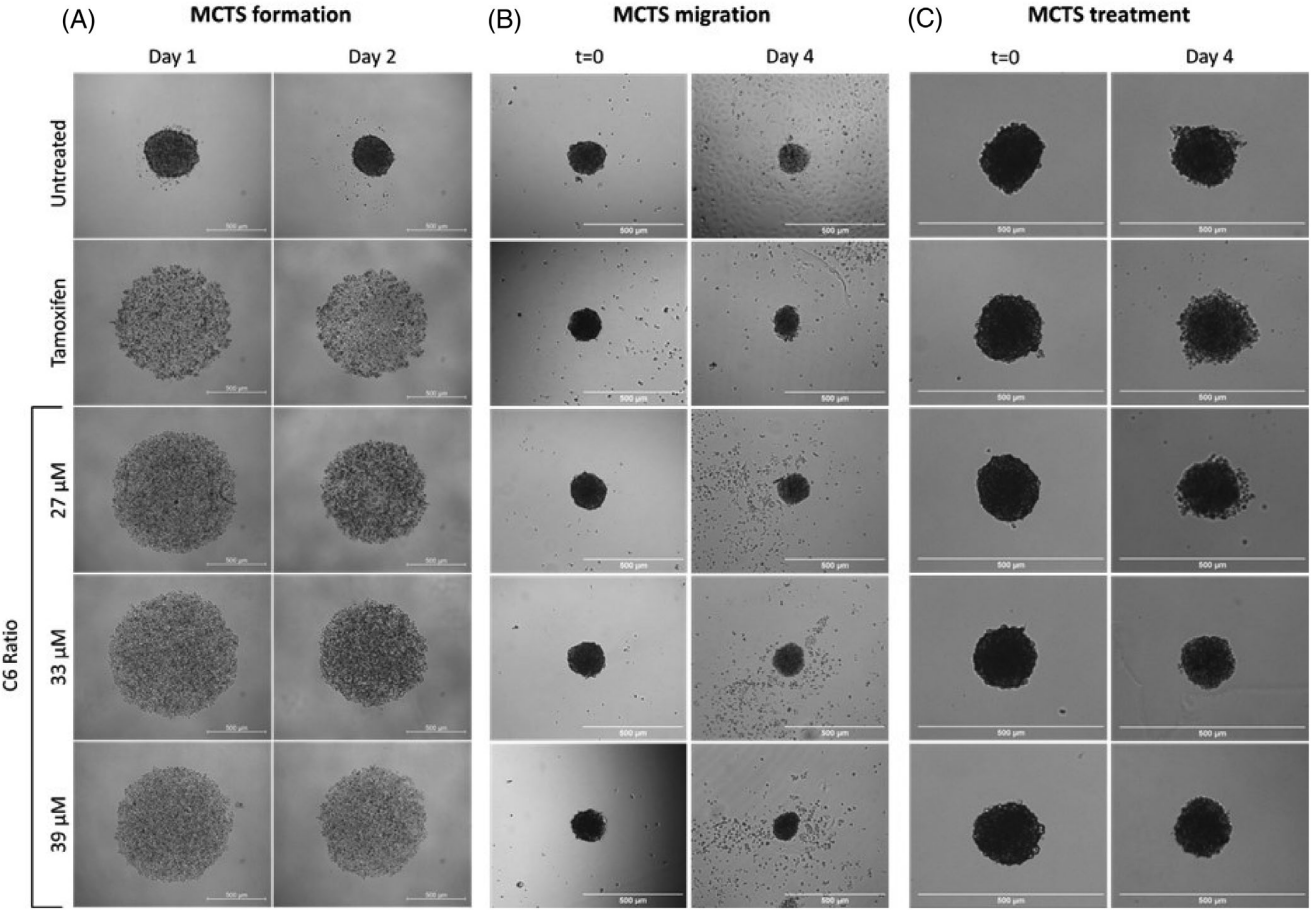
The effect of the cannabinoid ratio on MCF7 spheroid formation, migration, and treatment. The effect of treatment with tamoxifen (25 μM) and the cannabinoid ratio on spheroid (A) formation, (B) migration, and (C) size and density, determined using light microscopy. MCTS, multicellular tumour spheroid.

The untreated cells formed a dense spheroid by Day 1 of incubation, which decreased in size and increased in density by Day 2 (Figure 13A), suggesting that cell‐to‐cell adhesion increased to form the dense MCTS. In contrast, cells treated with tamoxifen and all concentrations of the cannabinoid ratio did not form a dense spheroid, suggesting that treatment inhibited spheroid formation.

Regarding spheroid migration, the untreated MCTS showed a time‐dependent increase in cell attachment and migration, with cells covering the entire field of view by Day 4 (Figure 13B). In contrast, cells treated with tamoxifen and the ratio showed no cell attachment or migration, which suggested that the treatments effectively inhibited cell migration.

The size of the untreated spheroid remained relatively constant over the 4 days of observation (Figure 13C). The spheroid treated with Tamoxifen showed decreased cell‐to‐cell adhesion and cell detachment near the edges of the spheroid which resulted in an overall decrease in the size of the spheroid. This same trend was also seen after treatment with the lowest concentration of the cannabinoid ratio. The higher cannabinoid concentrations showed an overall decrease in the size and density of the MCTS with increased time, suggesting that the cannabinoid ratio effectively treated the MCTS.

These results show that the effect of the cannabinoid ratio on spheroid formation, migration, and size was comparable to the effects induced by the standard endocrine treatment, Tamoxifen. These results serve as a proof‐of‐concept experiment to demonstrate the anticancer potential of the cannabinoid ratio in a 3D tissue culture system, and possibly in an in vivo system.

In conclusion, this study showed that—despite a significant decrease in viability after 24 h of treatment—the cannabinoid ratio did not induce cell cycle arrest, apoptosis, necrosis, autophagy, or ferroptosis in the MCF7 cells. This suggested an alternative mechanism of cell death, which was confirmed to be paraptosis. The mechanism of paraptosis induction was shown to be primarily dependent on Ca^2+^‐mediated communication between the ER and mitochondria. It was found that paraptosis was most likely induced by the CBD‐mediated modulation of VDAC, resulting in increased Ca^2+^ flux from the ER to the mitochondria, leading to subsequent mitochondrial Ca^2+^ overload, ER stress, ER and mitochondrial swelling, and the impairment of mitochondrial metabolism.

The limitations of this study were that no other Ca^2+^ transporters that have been implicated in paraptosis induction were tested, such as inositol 1,4,5,‐triphosphate receptors (IP3Rs), ryanodine receptors (RyRs), and the mitochondrial calcium uniporter (MCU) which are involved in Ca^2+^ transport from the ER to the mitochondria. While the role of VDAC modulation was confirmed, there is a possibility that multiple channels are modulated to exert the dysregulation of Ca^2+^ signalling that was observed. Another limitation was that a general cytoplasmic Ca^2+^ stain was not used to measure changes in cytoplasmic Ca^2+^ as some natural compounds have been shown to induce increases in both cytosolic and mitochondrial Ca^2+^ levels.

## AUTHOR CONTRIBUTIONS

**A. de la Harpe**: Conceptualization; formal analysis; investigation; methodology; project administration; visualization; writing – original draft preparation. **N. Beukes**: Conceptualization; project administration; supervision; writing – review and editing. **C. Frost**: Conceptualization; funding acquisition; project administration; resources; supervision; writing – review and editing.

## CONFLICT OF INTEREST STATEMENT

The authors declare no conflicts of interest.

## Supporting information


**Video S1:** Morphological changes in MCF‐7 cells after treatment with the cannabinoid ratio.

**Video S2:** CHX inhibition of vacuole formation in cannabis treated MCF‐7 cells.

**Video S3:** DIDS inhibition of vacuole formation in cannabis treated MCF‐7 cells.

## Data Availability

All data is available data is published in the article.
